# Rapidly Progressive Dementia in an Elderly Male: Perseverance Becomes the Key to a Rare Diagnosis

**DOI:** 10.7759/cureus.47231

**Published:** 2023-10-17

**Authors:** Arundhati Negi, M. Mukhyaprana Prabhu

**Affiliations:** 1 Internal Medicine, Kasturba Medical College, Manipal, IND

**Keywords:** human prion disease, csf 14-3-3 protein, rare neurodegenerative disease, rapidly progressive dementia, creutzfeldt–jakob disease

## Abstract

Cognitive impairment in a patient with rapidly progressive dementia (RPD) develops faster than expected for a known dementia syndrome. It poses as a diagnostic challenge for the physician who must identify the diagnosis among a broad spectrum of differentials. Here, we discuss the case of a 60-year-old male who presented with a four-month history of progressive gait disturbance, incoherent talking, dysarthria, hand tremors, and new-onset bladder incontinence. Neurological examination revealed fast saccades, cerebellar dysarthria, hypertonia, and normal power in all four limbs, brisk reflexes, past pointing, intentional tremors, resting myoclonic jerks, and ataxic gait. Initial differentials of progressive paraneoplastic encephalitis, infectious encephalitis, and toxic encephalopathy were considered. However, the results of lumbar puncture and blood investigations-voltage-gated potassium channel (VGKC) antibody and N-methyl-D-aspartate (NMDA) receptor antibody, tumor markers, viral markers being negative and ammonia and lactate levels being normal led us to think of another possibility. With such rapidly progressive dementia, myoclonic jerks, extrapyramidal signs, and cerebellar signs, a diagnosis of Creutzfeldt-Jakob disease (CJD) was taken into consideration. A cerebrospinal fluid (CSF) sample was sent for CSF protein 14-3-3 quantification by enzyme-linked immunosorbent assay (ELISA) and came out positive. During his stay in the hospital, our patient developed multiple complications, and his clinical state progressively worsened. With no signs of improvement and the known fatal nature of the disease, the goals of care were discussed with the family and we all agreed on providing palliative care. The patient passed away on day 15 of hospital admission.

## Introduction

Dementia is a slow, progressive neurological condition that impairs motor, cognitive, and behavioral function and ultimately results in functional impairment. Alzheimer's dementia (AD), vascular dementia (VD), frontotemporal dementia (FTD), and dementia with Lewy bodies (DLB) are common instances of chronic neurodegenerative disorders that lead to dementia. The designation of dementia as rapidly progressive dementia (RPD) is warranted in some cases where a subgroup of illnesses exists that exhibit a much faster deterioration in function [1]. Among the clinical conditions presenting with RPD, the most concerning differential diagnosis is Creutzfeldt-Jakob disease (CJD) - a rare, fatal, neurodegenerative disorder that occurs in humans - and is caused by the formation and transmission of pathological prion protein [2]. Here, we discuss the case of rapidly progressive dementia presenting in a 60-year-old male with underlying rheumatic heart disease and undergoing anticoagulation therapy. A thorough clinical workup was performed, and the results of investigations, including magnetic resonance imaging (MRI), electroencephalogram (EEG), and cerebrospinal fluid (CSF) analysis, led us to a diagnosis of probable CJD.

## Case presentation

A 60-year-old male, with a past medical history significant for childhood poliomyelitis, rheumatic heart disease status post-mitral valve replacement (nine years ago), carcinoma pyriformis status post-radiotherapy/chemotherapy (two years ago) presented to our hospital with a four-month history of swaying while walking. It was initially side to side and progressed to front and back as days progressed. He was able to feel his feet on the ground and could wear his footwear without slippage. He was able to feel his clothes on his body and feel hot and cold sensations as well. Motor deficits were present in the form of difficulty in bending knees and difficulty in lifting legs to climb stairs. These symptoms gradually worsened to an extent such that he had to drag both feet while walking. For the past three weeks before the presentation, the patient was unable to eat food from his own hands because of tremors in his hands. Tremors were present in both hands. He developed difficulty in swallowing with the presence of cough on swallowing, bladder incontinence, worsening of speech in the form of decreased word output, incoherent talking, and incomprehensible words. During his illness, he also had jerky movements and gait difficulty. Since his chemotherapy/ radiotherapy (two years ago), he had dysarthria, but a worsening of the same was noticed by the family members in the past three weeks. No family history of dementia was reported. Current medications of the patient include oral digoxin 0.25 mg once a day and oral acenocoumarol 1 mg once a day. On examination, the patient was confused and often not obeying commands. There was an impairment of recent and immediate memory. Mini-mental state examination could not be completed for our patient. Vertical eye movements were impaired. Bilateral gag reflex was decreased with dysarthria and nasal twang in speech. The tongue was midline with tremors present. The tone was increased in all four limbs. Power was assessed and was grade 5 (Medical Research Council grading) in both upper limbs and lower limbs, except toe flexion and extension, which was grade 3. Biceps reflex, triceps reflex, and knee reflex were brisk; ankle reflex was absent bilaterally; and plantar flexion was noted. Truncal ataxia, past pointing, fast saccades, and low amplitude resting myoclonic jerks were noted. Sensory system examination was normal, and there were no signs of meningeal irritation. Romberg’s test could not be performed.

Given the abovementioned history and clinical examination, the following differentials were taken into consideration - autoimmune encephalitis, progressive paraneoplastic encephalitis, vascular dementia, infectious encephalitis, toxic-metabolic encephalopathy, and neurodegenerative disease.

Blood investigations revealed normal thyroid hormones, vitamin B12, folic acid, urea, creatinine, and ammonia (plasma) levels. Blood cell counts were normal. There were no electrolyte abnormalities. The autoimmune encephalitis panel (VGKC and NMDA) was found to be negative. Tumor markers-carbohydrate antigen 19-9 (CA 19-9), cancer antigen 125 (CA 125), and alpha-fetoprotein (AFP)-were within normal range. Serology was negative for human immunodeficiency virus (HIV). hepatitis B surface antigen, antibodies to hepatitis C virus, and treponema pallidum hemagglutination test (TPHA) were negative. A strong positive Ro-52 marker was present on the antinuclear antibody (ANA) profile test. Antineutrophil cytoplasmic antibodies (ANCA) test was negative. Coagulation parameters, such as prothrombin time (PT)/international normalized ratio (INR) and activated partial thromboplastin time (APTT) were raised. Blood culture and urine culture were negative.

A lumbar puncture was performed after optimization of PT/INR. CSF was reported to be negative for cytomegalovirus (CMV), Japanese encephalitis virus (JEV), varicella zoster virus (VZV), herpes simplex virus (HSV), GeneXpert test for Mycobacterium tuberculosis, and India Ink staining for cryptococcus, ruling out the common etiology of infectious encephalitis (Table 1). CSF analysis did not reveal any abnormalities (Table 2). No atypical or malignant cells were seen on CSF microscopy. CSF culture was negative.

**Table 1:** CSF Analysis CSF: Cerebrospinal fluid DNA: Deoxyribonucleic acid IgM: Immunoglobulin M

TEST	RESULT
HSV	Negative
VZV	Negative
Anti-JEV IgM	Negative
CMV (DNA)	Negative
GeneXpert test for Mycobacterium tuberculosis	MTB not detected

**Table 2:** CSF characteristics

CSF Characteristics	Value
Color	Clear
CSF opening pressure (mm H_2_O)	200
Total WBC count	0 cells/ mm^3^
Total RBC count	3 cells/mm^3^
Glucose (mg/dL)	65
Protein (mg/dL)	33
Chloride (mmol/L)	129
Lactate (mg/dL)	16.7
Adenosine deaminase (U/L)	0.79

**Figure 1:**
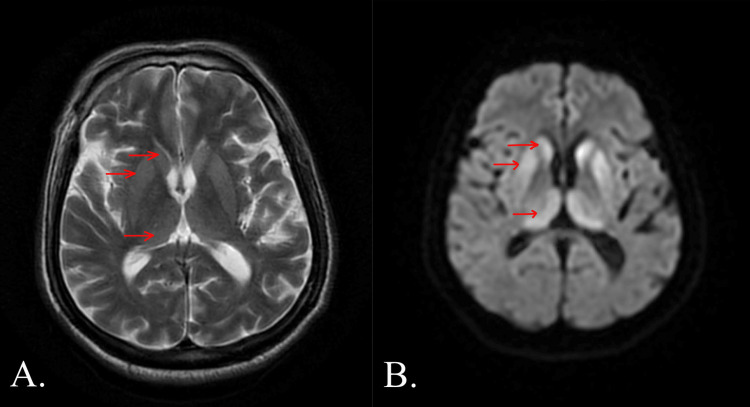
Non-contrast enhanced MRI BRAIN: A. Axial T2-weighted image and B. Axial DWI image, with arrows showing near-symmetric hyperintensities showing diffusion restriction noted in bilateral caudate lobes, anterior aspect of putamen and dorsomedial thalami MRI: Magnetic resonance imaging DWI: Diffusion-weighted imaging

A non-contrast-enhanced MRI study of the brain was requested and revealed near symmetric T2/fluid-attenuated inversion recovery (FLAIR) hyperintensities showing diffusion restriction noted in bilateral caudate lobes, anterior aspect of putamen and dorsomedial thalami (Figure 1). An EEG revealed continuous periodic sharp wave complexes (PSWC) with generalized slow waves.

The clinical features, progression of the disease course, suggestive MRI findings, and characteristic EEG findings led us to think of the possibility of Creutzfeldt-Jakob disease.

CSF sample was sent to an outside center for CSF protein 14-3-3 quantification, and the report came out to be positive.

While being admitted to the hospital, our patient developed sudden onset shortness of breath and tachycardia. Atrial fibrillation with a fast ventricular rate was noted on an electrocardiogram (ECG). In view of the abovementioned ECG changes, an amiodarone infusion was started which was followed by oral amiodarone. The patient was intubated owing to respiratory distress. Meropenem was started as the empirical antibiotic, which was then changed to cefazolin after sputum cultures grew methicillin-sensitive staphylococcus aureus. Unfortunately, his condition did not improve. Sepsis parameters worsened, and ventilator parameters declined further despite showing initial improvement.

Given the patient's existing multiple comorbidities, lack of clinical improvement, and the known fatal outcome of probable CJD, we discussed the goals of care with the patient's relatives, and the decision to provide palliative care was agreed upon. The patient passed away on the 15th day of his hospital stay.

## Discussion

“Rapidly progressive dementia” (RPD) is a clinical scenario characterized by cognitive decline, which progresses into the clinical syndrome of dementia, within a relatively short period of time. This period is commonly considered to be less than either 1 or 2 years. The majority of patients develop significant functional impairment and progress to complete (or near-complete) dependence. RPD accounts for 3%-4% of dementia cases seen in clinical practice [3]. A difference noted between chronic dementias and RPD, apart from the time taken in disease progression, is in the prognosis of these two. While the former is known to have a more well-defined and known prognosis, the latter has a variable prognosis and is dependent on the underlying cause [1].

RPD encompasses a broad range of differentials, including vascular, autoimmune, progressive paraneoplastic encephalitis, viral encephalitis, progressive multifocal leukoencephalopathy, HIV dementia, neurodegenerative conditions, fungal infections, metastasis/neoplasm, and iatrogenic causes [4]. It presents a challenge to the physician who must differentiate possibly reversible processes like paraneoplastic disease (PND), Hashimoto's encephalitis (HE), and infectious or autoimmune etiologies from an irreversible neurodegenerative condition like CJD [1].

Our patient presented with clinical features of RPD and fulfilled the MRI-CJD Consortium diagnostic criteria, as a probable sporadic Creutzfeldt-Jakob disease (sCJD) case during the antemortem stage based on the clinical findings, positive MRI, EEG findings, and CSF analysis [5,6].

Transmissible spongiform encephalopathies (TSEs) are progressive neurodegenerative diseases that have a long incubation period ranging from several months to many years. CJD is the most common TSE affecting humans [4]. Patients show a rapidly deteriorating clinical course and a median survival of a mere 3.5-6 months after symptom onset [7]. CJD has an annual incidence of 1-2 cases per million population all over the world [2]. In India, the disease is extremely rare with a reported 0.01 cases per 1,000,000 population per year [8]. A study has found that individuals with a family history of dementia, a past history of poliomyelitis, those working in healthcare, and those who had previous contact with cows and sheep exhibited a heightened risk of developing CJD [9].

Based on the etiology, CJD has four subtypes: sporadic (85-90%), familial, iatrogenic, and variant. Sporadic CJD is the most common subtype. The average age of onset of sCJD is 65 years [2]. Sporadic CJD is caused by a spontaneous conformational change of normal cellular prion protein (PrPc) into its pathological form (PrPSc). This abnormal scrapie of prion protein accumulates in the brain and leads to spongiform degeneration and gliosis [8]. Research shows that the prion protein has high selectivity to copper ion (Cu2+) and functions as a neuroprotectant. Ablation of the normal prion protein has resulted in a damaging effect on the peripheral nervous system by triggering demyelinating neuropathy. In the central nervous system, it has been shown to be involved in maintaining sleep, memory, and neuroprotection [10].

CJD has a heterogenous clinical presentation. It manifests as rapidly progressive dementia associated with neuropsychiatric symptoms, myoclonus (most common sign), visual symptoms, akinetic mutism, cerebellar signs, and extrapyramidal signs. It has been found that approximately one-third of sCJD cases initially present with cognitive or behavioral changes, and close to one-third have cerebellar ataxia, aphasia, visual dysfunction, and motor deficit as the initial presenting symptoms. Depression has also been found in some patients with CJD [11]. Myoclonus can sometimes be absent initially, but present in the advanced stages of sCJD [12]. RPD with an average duration of as little as six months has been seen as a constant finding. Patients invariably developed coma and succumbed to the illness over several months [13].

Because of its highly varied initial clinical presentation and very low incidence, diagnosing CJD can be challenging. Other rapidly progressive dementias have overlapping neurocognitive and psychiatric manifestations, which can lead to added difficulties [12]. Apart from a thorough clinical history and examination, an extensive diagnostic workup that involves radiological investigations, vascular imaging studies, EEG studies, CSF analysis, blood, and serological studies are used to arrive at the diagnosis [4].

Histopathological confirmation is the gold standard investigation for definitive diagnosis [4]. Several diagnostic tests can be performed on antemortem. MRI studies of patients with sporadic CJD reveal hyperintense signals on DWI, FLAIR, and T2-weighted images involving the cerebral cortex, corpus striatum, putamen, and caudate. EEG shows characteristic periodic synchronous bi- or triphasic periodic sharp wave complexes (PSWC). CSF analysis of patients can be done for the detection of 14-3-3 protein and tau protein levels, of which the latter is more specific and has higher accuracy. Additional investigations in CJD involve the use of cerebrospinal fluid real-time quaking-induced conversion (CSF RT-QuIC), which has a sensitivity ranging from 87% to 91% [14].

## Conclusions

Patients with RPD typically develop dementia over weeks to months, in contrast with the slowly progressive dementias occurring over a few years. Due to the rapid clinical deterioration in RPD patients, urgent evaluation is essential and often requires a thorough workup, involving multiple tests being sent or performed concurrently. For any patient presenting with RPD, myoclonus, and cerebellar signs, CJD should be included among the differentials. However, it is important to first rule out other more common etiologies of RPD such as vascular, infectious, toxic-metabolic, autoimmune, neoplastic, iatrogenic, neurodegenerative, and structural causes before suspecting this rare diagnosis. Apart from a thorough clinical history and examination, investigations such as MRI studies, EEG studies, and CSF analysis are crucial for diagnosing CJD. Only supportive and palliative care can be provided to CJD patients as this disease is invariably fatal.
